# Resolutions Entered into by the Grand Jury, at Horsham, the 19th of March, 1804

**Published:** 1804-07-01

**Authors:** John Thomas Capel

**Affiliations:** Foreman of the Grand Jury


*Resolutions entered into by the Grand Jury, at Horsham, the 19th of March, 1804.*

The Honourable JOHN THOMAS CAPEL,
FOREMAN of the GRAND JURY.

RESOLVED,

1. That the Grand Jury do highly approve of any attempt which may be made, to suppress and eradicate that most fatal distemper the small-pox, and are willing, individually, and as far as their influence extends, to further the views of the Royal Jennerian Institution, already established in London for that purpose.

2. That the Grand Jury do strongly recommend to all medical persons, and others, of the county of Sussex, immediately to adopt and encourage the Jennerian system of inoculation, and do request them to transmit such plans on or before the 15th of April, as may appear most conducive to attain the desired object of eradicating the small-pox, to the person who by the Committee hereinafter-mentioned, may appear best qualified for the office of Secretary, and which appointment the Committee are requested to notify in the public prints of the county, as soon as it has taken place.

3. That the undermentioned noblemen and gentlemen, who signed the recommendation of the Jennerian system of inoculation, at the meeting of Deputy Lieutenancy at Lewes, together with the county Members and Foreman of the Grand Jury, be requested to form the Committee, with any other county gentlemen who may be desirous of encouraging this praise-worthy institution.

Names of the Committee:

| | |
|---|---|
| Lord Pelham, | George Shiffner, Esq. |
| Lord Gage, | Thomas Partington, Esq. |
| Lord Sheffield, | General Lenox, } County |
| Sir J. Bridger, | John Fuller, Esq. } Members. |
| Thomas Kemp, Esq. | Hon. John Tho. Capel. |

4. That the thanks of the Grand Jury be given to Dr. Tierney, of Brighton, for his plan delivered in for promoting Vaccine Inoculation.

5. That a subscription be entered into to defray the present expences, as well as for all other purposes intended to be promoted by this undertaking.

6. That the above resolutions be printed in the Lewes Journal, Portsmouth Telegraph, and two London Papers.

7. That John Drew, of Chichester, Esq.; Francis Whitfield, Esq. of Lewes; and Tilden Sampson, Esq. of Battle, be requested to receive subscriptions for the use of the institution.

